# Chylothorax Secondary to Behçet’s Disease: A Case Report

**DOI:** 10.7759/cureus.115792

**Published:** 2026-09-04

**Authors:** Ferdaous Kasmi, Karima Marc

**Affiliations:** 1 Department of Pulmonology, Moulay Youssef Hospital, Ibn Sina University Hospital, Rabat, MAR

**Keywords:** behçet's disease, chylothorax, cyclophosphamide, pleural effusion, superior vena cava thrombosis, vasculitis

## Abstract

Chylothorax is an uncommon cause of pleural effusion and is most frequently associated with trauma or malignancy. Its occurrence in Behçet's disease (BD) is exceptionally rare and is usually related to extensive central venous thrombosis causing obstruction of the thoracic duct.

We report the case of a 35-year-old woman with a five-year history of BD who presented with progressive dyspnea, dry cough, and mild hemoptysis. Chest computed tomography (CT) revealed extensive thrombosis of the superior vena cava extending to both brachiocephalic veins and the right subclavian vein, associated with large bilateral pleural effusions. Diagnostic thoracentesis yielded milky pleural fluid with elevated triglyceride levels, confirming bilateral chylothorax. Other potential causes, including pulmonary embolism, pulmonary artery aneurysm, malignancy, chylopericardium, and chyloperitoneum, were excluded. The patient was treated with therapeutic anticoagulation, high-dose corticosteroids, intravenous cyclophosphamide, repeated therapeutic thoracentesis, and nutritional support with dietary fat restriction. Clinical and radiological outcomes were favorable, with complete resolution of dyspnea and marked regression of the pleural effusions.

Chylothorax is a rare but important thoracic manifestation of BD. In patients presenting with unexplained pleural effusion and central venous thrombosis, BD should be considered among the differential diagnoses. Early recognition and prompt immunosuppressive therapy, combined with appropriate management of the pleural effusion, are essential to achieve favorable outcomes.

## Introduction

Pleural involvement is an uncommon but recognized manifestation of connective tissue diseases (CTDs) and systemic vasculitides. It occurs most frequently in systemic lupus erythematosus (30-60%) and rheumatoid arthritis (5-20%), whereas it remains rare in Behçet's disease (BD), in which thoracic involvement affects approximately 1-8% of patients and pleural manifestations account for only a small proportion of these cases [1-4]. In BD, pleural effusion is usually secondary to vascular complications, particularly thrombosis of the superior vena cava or major thoracic veins, pulmonary artery aneurysms, or pulmonary infarction. Chylothorax is an exceptionally rare manifestation resulting from thoracic duct obstruction caused by extensive venous thrombosis [4-6]. The interval between the diagnosis of BD and pleural involvement is highly variable, ranging from the initial presentation to several years after disease onset, although most reported cases occur in patients with longstanding vascular disease [5-7]. Early recognition is essential, as prompt immunosuppressive therapy, combined with appropriate management of the pleural complication, is crucial for controlling the underlying vasculitic process and improving clinical outcomes [4-7].

We report the case of a 35-year-old woman with a five-year history of BD who presented with bilateral chylothorax secondary to extensive central venous thrombosis.

## Case presentation

A 35-year-old woman with a five-year history of BD, treated with colchicine and dapsone, presented with a two-month history of progressively worsening dyspnea, initially on exertion and subsequently at rest. Her symptoms were associated with a dry cough and one episode of mild hemoptysis, occurring in the absence of fever but accompanied by asthenia, anorexia, and unintentional weight loss.

On admission, the patient was tachypneic, with an oxygen saturation of 87% on room air, which improved with supplemental oxygen at 5 L/min. Physical examination revealed bilateral lower-limb edema, diffuse pseudofolliculitis involving the trunk, face, and extremities, and clinical signs of bilateral pleural effusions, more pronounced on the right.

Chest radiography demonstrated bilateral pleural effusions (Figure 1). Contrast-enhanced computed tomography (CT) of the chest revealed extensive thrombosis of the superior vena cava extending into both brachiocephalic veins and the right subclavian vein. No pulmonary embolism or pulmonary artery aneurysm was identified. Large right-sided and moderate left-sided pleural effusions were also observed.

**Figure 1:**
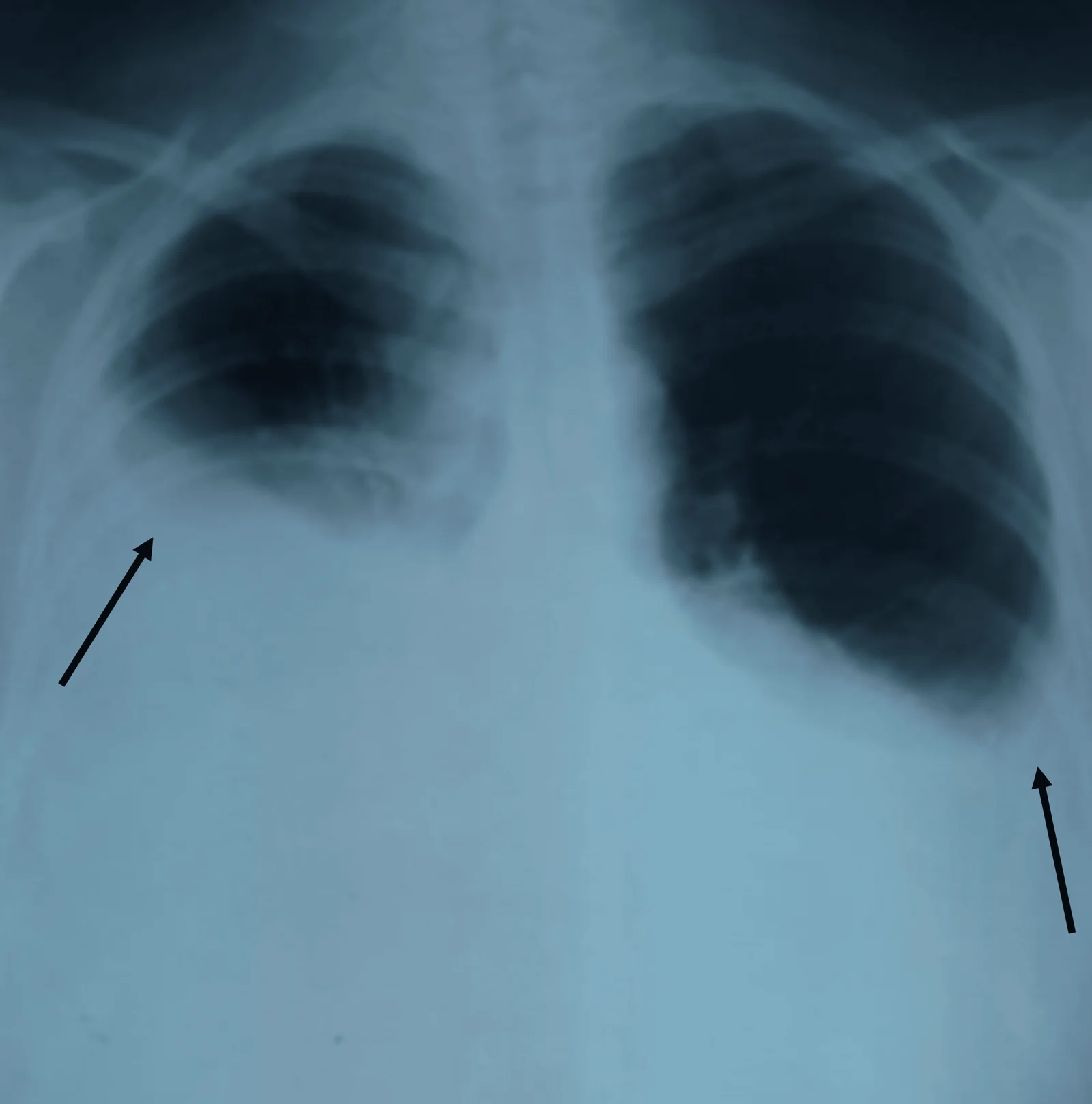
Chest radiograph showing bilateral pleural effusion, with blunting of the costophrenic angles. The left arrow indicates the right pleural effusion, while the right arrow indicates the left pleural effusion.

Laboratory investigations revealed leukocytosis (12.0 × 10⁹/L) with marked neutrophilia (11.0 × 10⁹/L). Hemoglobin was 11.0 g/dL, while the platelet count was within the normal range. C-reactive protein (CRP) was elevated at 20 mg/L. Renal function, serum electrolytes, and liver function tests, including aspartate aminotransferase (AST), alanine aminotransferase (ALT), alkaline phosphatase (ALP), gamma-glutamyl transferase (GGT), total bilirubin, serum albumin, and total protein, were all within normal limits. Serological tests for hepatitis B virus, hepatitis C virus, and human immunodeficiency virus were negative. Serum protein electrophoresis revealed no monoclonal gammopathy or other significant abnormalities, with a normal albumin-to-globulin ratio. Diagnostic thoracentesis yielded a milky pleural fluid (Figure 2). Biochemical analysis confirmed chylothorax, with a pleural fluid protein concentration of 21 g/L, cholesterol of 0.44 g/L, and triglyceride level of 2.97 g/L. The results are presented in Table 1.

**Figure 2:**
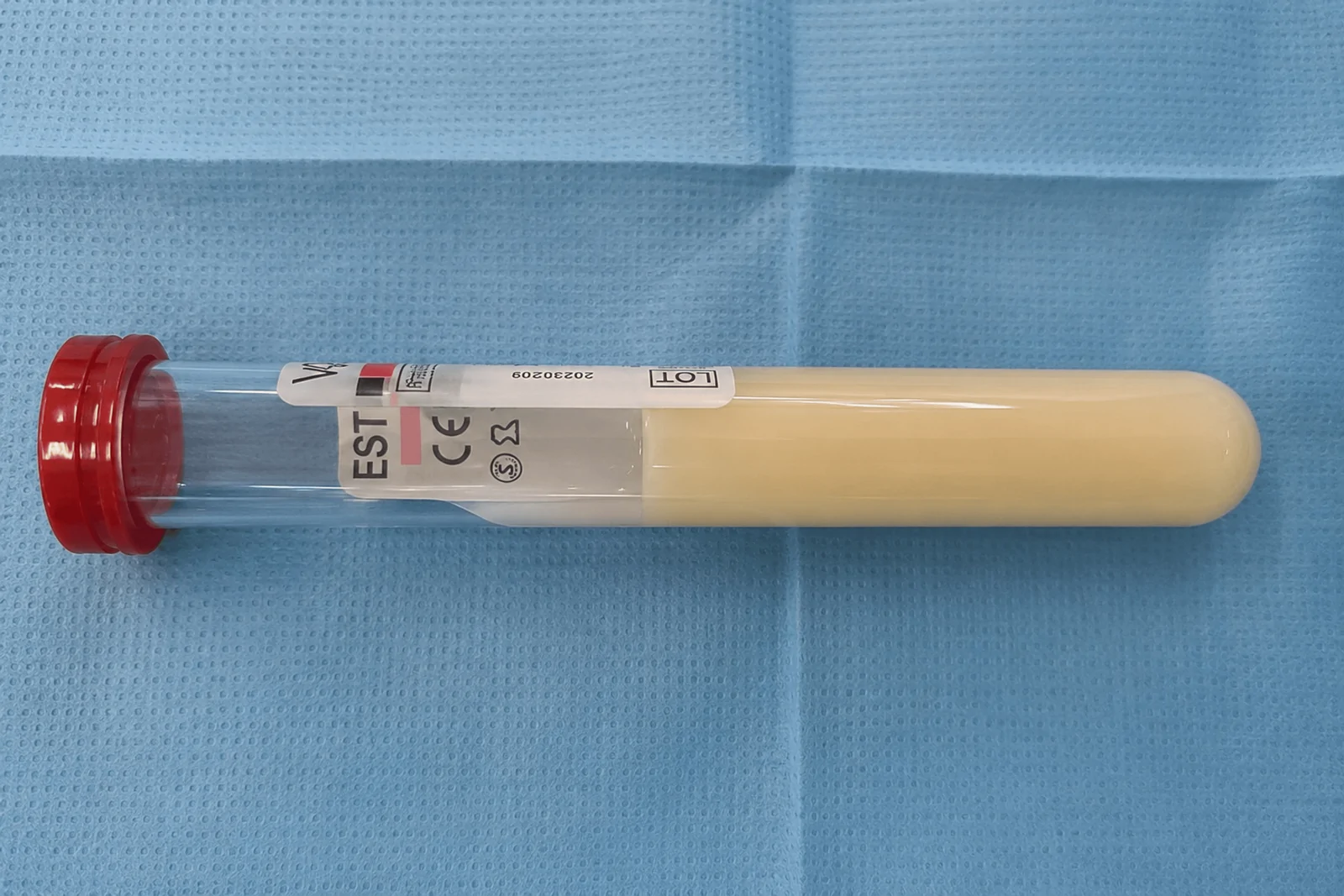
Characteristic milky appearance of the chylous pleural fluid.

**Table 1:** Laboratory and pleural fluid findings. AST: aspartate aminotransferase; ALT: alanine aminotransferase; ALP: alkaline phosphatase; GGT: gamma-glutamyl transferase

Parameter	Patient value	Reference range	Unit
White blood cell count (WBC)	12.0	4.0–10.0	×10⁹/L
Neutrophils	11.0	1.5–7.5	×10⁹/L
Hemoglobin	11.0	12–16	g/dL
Platelet count	Within normal range	150–400	×10⁹/L
C-reactive protein (CRP)	20	<5	mg/L
Pleural fluid protein	21	No established diagnostic cutoff	g/L
Pleural fluid cholesterol	0.44	<2.0	g/L
Pleural fluid triglycerides	2.97	<1.24	g/L
Renal function	Within normal range	Laboratory-specific	—
Serum electrolytes	Within normal range	Laboratory-specific	—
Liver function tests (AST, ALT, ALP, GGT, bilirubin)	Within normal range	Laboratory-specific	—
Serum albumin	Within normal range	Laboratory-specific	g/L
Total protein	Within normal range	Laboratory-specific	g/L

Abdominal ultrasonography showed no evidence of ascites or chylous peritoneal effusion (chyloperitoneum). Transthoracic echocardiography revealed normal biventricular systolic function, with no evidence of pulmonary hypertension or pericardial effusion, including chylopericardium.

The patient was treated with therapeutic anticoagulation, high-dose corticosteroids, dietary fat restriction with nutritional support, and bilateral therapeutic thoracentesis, draining 1,700 mL from the right pleural cavity and 900 mL from the left. Monthly intravenous cyclophosphamide therapy was subsequently initiated.

The clinical course was favorable after two weeks, with resolution of dyspnea, improvement in oxygenation, and marked regression of the bilateral pleural effusions on follow-up chest radiography (Figure 3).

**Figure 3:**
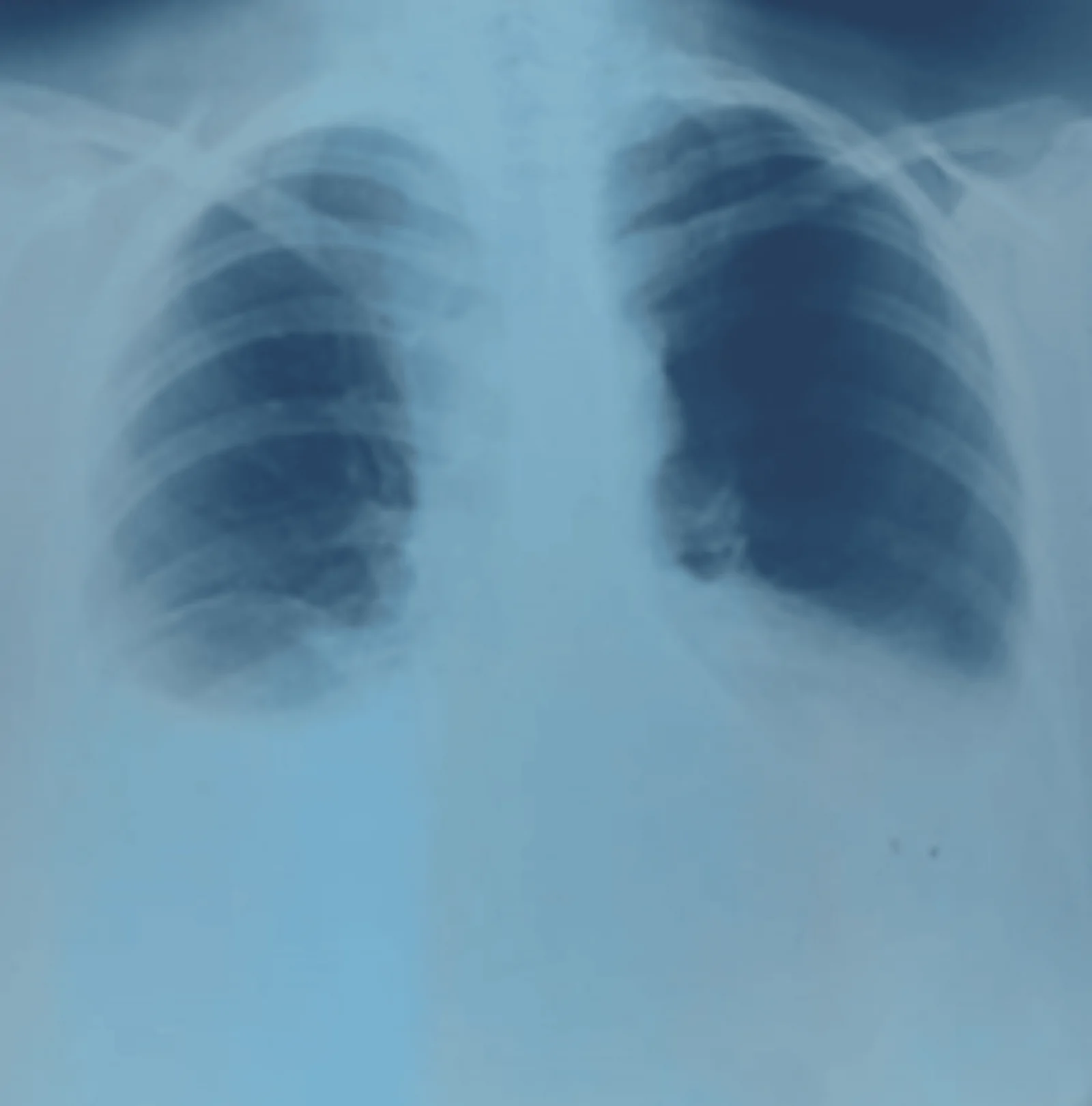
Chest X-ray after therapeutic thoracentesis.

## Discussion

Pleural involvement is a rare but well-documented manifestation of CTDs and systemic vasculitides. It is most commonly observed in systemic lupus erythematosus (30-60%) and rheumatoid arthritis (5-20%), but is uncommon in BD, where thoracic involvement occurs in approximately 1-8% of patients and pleural manifestations constitute only a small subset of these cases [1-4].

BD is a chronic relapsing multisystem vasculitis characterized by recurrent oral and genital ulcers, ocular inflammation, and variable vascular, neurological, and gastrointestinal involvement. Thoracic manifestations are uncommon, occurring in approximately 1-8% of patients, and mainly include pulmonary artery aneurysms, venous thrombosis, pulmonary infarction, and, less frequently, pleural involvement [8,9]. Pleural effusion is an unusual manifestation of BD and is generally secondary to vascular complications rather than primary pleural inflammation. Chylothorax represents one of the rarest thoracic manifestations, with only a limited number of cases reported in the literature [8,9].

The pathogenesis of chylothorax in BD is closely related to the disease's characteristic venous vasculitis. Inflammation of large veins predisposes to thrombosis, particularly involving the superior vena cava, brachiocephalic veins, and subclavian veins. Obstruction of these central venous structures impairs thoracic duct drainage, resulting in increased lymphatic pressure, rupture of lymphatic vessels, and leakage of chyle into the pleural cavity [6,8,9]. In the present case, extensive thrombosis of the superior vena cava extending to both brachiocephalic veins and the right subclavian vein, in the absence of pulmonary embolism or pulmonary artery aneurysm, strongly supports this mechanism.

The diagnosis of chylothorax relies on pleural fluid analysis. A milky appearance is highly suggestive but not pathognomonic. The diagnosis is confirmed by a pleural fluid triglyceride concentration greater than 110 mg/dL (1.24 mmol/L), whereas values below 50 mg/dL virtually exclude chylothorax. Intermediate values require lipoprotein electrophoresis to demonstrate chylomicrons, which remains the diagnostic gold standard [1,2]. Once chylothorax is established, traumatic causes, malignancy, particularly lymphoma, congenital lymphatic disorders, infections such as tuberculosis, central venous thrombosis, and autoimmune diseases such as systemic lupus erythematosus and antiphospholipid syndrome should be systematically excluded before attributing the condition to BD [3,4]. In our patient, the absence of trauma, surgery, malignancy, pulmonary embolism, pulmonary artery aneurysm, chyloperitoneum, and chylopericardium, together with extensive central venous thrombosis related to BD, strongly supported the diagnosis of secondary chylothorax.

The interval between the diagnosis of BD and the development of chylothorax is highly variable, ranging from the initial presentation to several years after disease onset. Most reported patients had longstanding disease complicated by major venous thrombosis, similar to our patient, who developed chylothorax five years after the diagnosis of BD [5-7]. This variability highlights the importance of maintaining a high index of suspicion in patients with BD presenting with unexplained pleural effusion and symptoms suggestive of superior vena cava obstruction.

Management of chylothorax associated with BD should address both the pleural effusion and the underlying inflammatory vasculitis. Therapeutic thoracentesis or chest drainage provides symptomatic relief, while dietary fat restriction with medium-chain triglycerides or total parenteral nutrition may reduce chyle production [9]. However, definitive treatment relies on aggressive immunosuppressive therapy aimed at controlling the underlying vasculitic process. High-dose corticosteroids combined with cyclophosphamide remain the preferred regimen in patients with severe vascular involvement, whereas azathioprine, mycophenolate mofetil, or anti-tumor necrosis factor agents may be considered in refractory or relapsing disease [8,9]. The use of anticoagulation remains controversial because venous thrombosis in BD results primarily from inflammation rather than hypercoagulability. Nevertheless, anticoagulation may be considered on an individual basis after excluding pulmonary artery aneurysms because of the potential risk of life-threatening hemoptysis [8-10].

Our patient showed rapid clinical and radiological improvement following therapeutic thoracentesis, nutritional management, corticosteroids, cyclophosphamide, and anticoagulation, supporting previous reports that successful treatment depends primarily on early recognition of the inflammatory vascular process and prompt initiation of immunosuppressive therapy. Although exceptionally rare, chylothorax should therefore be considered in the differential diagnosis of pleural effusion in patients with BD, particularly in the presence of central venous thrombosis.

## Conclusions

Chylothorax is an exceptionally rare but potentially serious thoracic manifestation of BD. It should be suspected in patients presenting with unexplained pleural effusion associated with central venous thrombosis or other features suggestive of vascular BD. Early recognition, prompt pleural fluid analysis, and appropriate imaging are essential to establish the diagnosis and exclude more common causes of chylothorax. Management should target both the pleural effusion and the underlying inflammatory vasculitis through a multidisciplinary approach combining nutritional support, pleural drainage when indicated, and aggressive immunosuppressive therapy. Our case highlights the importance of considering BD in the differential diagnosis of non-traumatic chylothorax and demonstrates that early immunosuppressive treatment can lead to a favorable clinical outcome.
